# Social Power Increases Interoceptive Accuracy

**DOI:** 10.3389/fpsyg.2017.01322

**Published:** 2017-08-03

**Authors:** Mehrad Moeini-Jazani, Klemens Knoeferle, Laura de Molière, Elia Gatti, Luk Warlop

**Affiliations:** ^1^Department of Marketing, Faculty of Economics and Business, University of Groningen Groningen, Netherlands; ^2^Center for Multisensory Marketing, Department of Marketing, BI Norwegian Business School Oslo, Norway; ^3^Cognitive, Perceptual, and Brain Sciences, University College London London, United Kingdom; ^4^Multisensory Experiences (Informatics) Psychology and Brain Sciences, University of Sussex Brighton, United Kingdom

**Keywords:** social power, interoceptive accuracy, self-focused attention, body-consciousness, sense of power

## Abstract

Building on recent psychological research showing that power increases self-focused attention, we propose that having power increases accuracy in perception of bodily signals, a phenomenon known as interoceptive accuracy. Consistent with our proposition, participants in a high-power experimental condition outperformed those in the control and low-power conditions in the Schandry heartbeat-detection task. We demonstrate that the effect of power on interoceptive accuracy is not explained by participants’ physiological arousal, affective state, or general intention for accuracy. Rather, consistent with our reasoning that experiencing power shifts attentional resources inward, we show that the effect of power on interoceptive accuracy is dependent on individuals’ chronic tendency to focus on their internal sensations. Moreover, we demonstrate that individuals’ chronic sense of power also predicts interoceptive accuracy similar to, and independent of, how their situationally induced feeling of power does. We therefore provide further support on the relation between power and enhanced perception of bodily signals. Our findings offer a novel perspective–a psychophysiological account–on how power might affect judgments and behavior. We highlight and discuss some of these intriguing possibilities for future research.

“*I rely far more on gut instinct than researching huge amounts of statistics*.” – Richard Branson, founder of Virgin Group, business magnate, investor, and philanthropist.

## Introduction

In interviews and autobiographies, the powerful, be they high-ranked military commanders, well-known politicians, or business magnates, recurrently emphasize the role of gut feelings in their decisions. For instance, in an interview, George W. Bush, 43rd President of United States, described himself as “a gut player” who relied largely on his instinct when making critical decisions in the aftermath of September 11 attacks (Woodward, 2002). Powerholders’ tendency to rely on their gut feelings goes beyond the anecdotal evidence. For example, in its recent survey of 174 executives around the world, the Economist Intelligence Unit (EIU) found that 9 in every 10 executives have the tendency to ignore or reanalyse the data if it contradicts their gut feelings and intuitions (Bird and Swabey, 2014). Together, these pieces of evidence suggest that having power increases reliance on and sensitivity to internal signals. The question, however, is whether the powerful do this because they might be better at sensing those signals than the average person might be. In other words, power might enhance the perception of bodily signals, a capacity known as interoceptive accuracy (Craig, 2002). Investigating the relation between social power and interoceptive accuracy is important for at least two reasons. First, interoception plays a key role in shaping one’s bodily experiences and sense of self-awareness, the sense of being “me” (Craig, 2009; Damasio, 2010). Interoceptive accuracy intensifies the experience of emotions and visceral states (Wiens et al., 2000; Craig, 2004; Pollatos et al., 2005; Wiens, 2005; Herbert et al., 2007), and has been found to influence intuitive judgments and decision-making (Werner et al., 2009; Dunn et al., 2010). Therefore, the link between power and interoceptive accuracy can provide crucial insights as to how having power shapes a person’s experiences, decisions and behavior. Second, despite numerous findings in the past decade on how social power influences people’s cognition, emotion and behavior (for review, see Guinote, 2017), little is known about the psychophysiological effects of social power. Thus, exploring how power might affect interoceptive accuracy can advance our understanding of the basic processes through which power influences behavior.

In the present research, for the first time, we provide experimental evidence that the powerful, relative to people in the powerless and control conditions, are more accurate in perceiving their bodily signals (i.e., interoceptive accuracy). In the following, we first review psychological research showing that experiencing power liberates the self from external influences (e.g., social threats and environmental stressors) and shifts attentional resources inward. Building on these findings, we then propose that the powerholders’ unwavering self-focused attention enables them to perceive their bodily signals more accurately, than do people in the powerless and control conditions. Subsequently, we report the results of an extensive experiment designed to test this proposition. Finally, we discuss contributions of our research as well as its implications for future research.

## Social Power, Self-Focused Attention, and Interoceptive Accuracy

Power is key to understanding the dynamics of social relations and hierarchies in primate groups, both human and non-human (Fiske, 1992, 2010). Although power often correlates with social class and status (i.e., social respect), it differs from them in that power varies across situations more than socioeconomic status does. One advantage of the situational malleability of social power is that researchers can establish causal (and not just correlational) relations between power and desired outcome variables by experimentally manipulating power.

Power is defined as the asymmetrical control over valued resources (e.g., money, food, knowledge, etc.) in social relations (Fiske, 2010). Asymmetrical control over resources brings about asymmetrical dependencies between parties, wherein the powerful are less dependent on the powerless and determine the outcomes of the powerless (Emerson, 1962; Fiske, 1993). Higher access to valued resources and lower dependency on others reduce powerholders’ external and social concerns, enabling them to shift their attentional focus predominantly inward (Fiske and Dépret, 1996; Fiske, 2010; Keltner et al., 2003).

Supporting these arguments, a wealth of experimental research has shown that feeling powerful systematically decreases people’s attention to the external environment and reduces their motivation for affiliation and social engagement. For example, having power has been found to reduce perspective-taking, the ability to step outside one’s own experience and visualize the psychological states of others (Galinsky et al., 2006). In one striking experiment, when asked to draw the letter *E* on their foreheads, participants in a high-power experimental condition were more likely than those in a low-power condition to draw the *E* in a self-oriented way, as if reading it themselves (a backward *E* from another person’s perspective). Having power has also been found to reduce distress and compassion for others (Van Kleef et al., 2008). Similarly, in negotiation experiments, participants in the high-power experimental condition were less likely to notice and consider their opponents’ emotions, than were participants in the low-power condition (Van Kleef et al., 2004, 2006). Moreover, having power reduces motivation to gather information about others to form accurate impressions about them (Neuberg and Fiske, 1987) and increases people’s tendency to stereotype their subordinates (Goodwin et al., 2000).

Attention is a limited resource and interoceptive and exteroceptive stimuli compete for organisms’ limited information processing capacity (Pennebaker and Lightner, 1980; Pennebaker, 1982). By diminishing the motivation to attend to the external environment, having power facilitates shifting attention mainly inward, promoting a state of self-focused attention (Fiske, 2010). In line with this argument, past findings have shown that having power, relative to lack of it, bolsters the effects of internal processes (e.g., one’s own thoughts, feelings, and personality characteristics) on judgements and decisions. For example, in a negotiation task, participants in the high-power experimental condition were more influenced by their own social value orientation, than by their opponents’ reputation (Galinsky et al., 2008). Similar experimental evidence has shown that the powerful are more focused on and inspired by their own ideas and less influenced by other people’s ideas (Galinsky et al., 2008; Van Kleef et al., 2015). Moreover, having power has been found to increase self-projection, the tendency to refer to one’s own characteristics and internal states when judging other people’s states (Overbeck and Droutman, 2013). Research demonstrating that having power increases confidence in one’s own thoughts (Brinol et al., 2007) and decisions (Fast et al., 2009) provide further support for the idea that power boosts self-focused attention and increases reliance on internal states.

Building on the reviewed findings, we propose that one consequence of the unwavering self-focused attention among the powerful is the ability to perceive bodily signals more accurately, than people in the powerless and control conditions do. Our proposition is consistent with the *perceptual accuracy hypothesis* of the self-awareness theory, which suggests that factors shifting attentional resources inward should promote higher access to bodily signals and increase interoceptive accuracy (Gibbons et al., 1979; Scheier et al., 1979; Ehlers and Breuer, 1996; Carver, 2011).

Two paradigms have been commonly used to quantify interoceptive accuracy in laboratory settings. One paradigm is the Schandry heartbeat-detection task, also known as the Mental Tracking Method, which requires participants to silently count their felt heartbeats. The accuracy in perceiving heartbeats indicates one’s ability to detect bodily signals (Schandry, 1981; Dunn et al., 2010). The other paradigm–the Whitehead task–is based on discriminating between exteroceptive cues (e.g., auditory or visual) and visceral signals (Whitehead et al., 1977; Brener et al., 1993). In the Whitehead task, participants are asked to detect synchrony between a recorded heartbeat sound (i.e., exteroceptive audio signal) and their own heartbeats (i.e., interoceptive signal). Accuracy in detecting synchronous signals indicates one’s ability to detect bodily signals. Although these paradigms are both associated with activity in brain regions related to interoception (Critchley et al., 2004; Pollatos et al., 2007c), they impose different attentional demands on participants. Whereas the Schandry heartbeat-detection task only demands attention to visceral sensations, the Whitehead task requires focus on interoceptive (i.e., visceral sensations) and exteroceptive stimuli simultaneously. Therefore, while the Schandry task exclusively measures interoceptive accuracy, the Whitehead task reveals people’s ability for multisensory (interoceptive and exteroceptive) integration (Brener and Ring, 1995; Pollatos et al., 2007c; Schulz et al., 2013). Given that our hypothesis was pertained to people’s interoceptive accuracy (and not to their ability in multisensory integration), we used the Schandry heartbeat-detection task to test our proposition. In the following, we report the procedure and results of an experiment that examined the effect of situationally induced feeling of social power on interoceptive accuracy.

## Materials and Methods

### Participants

Participants were 135 paid university students (*M*_age_ = 23.74, *SD* = 4.14; 88 females). All participants gave written informed consent in accordance with the Declaration of Helsinki, and were fully debriefed afterward. The university’s research ethics committee approved the study and its procedure prior to data collection.

### Measuring Dispositional Characteristics

Two weeks before the laboratory experiment, participants completed a brief online questionnaire consisting of the private body-consciousness (PBC) scale (Miller et al., 1981), the personal sense of power (PSOP) scale (Anderson et al., 2012), and demographic questions (i.e., age and gender). The PBC scale captures participants’ chronic tendency to focus on bodily sensations. The PSOP measures people’s chronic and general sense of power, formed over time and across social contexts. We collected these measures to explore the role of dispositional characteristics (i.e., PSB and PSOP) in the relation between the situationally induced feeling of power (as manipulated in the lab) and interoceptive accuracy, and to test assumptions underlying our proposition. We measured these personality traits two weeks before the experiment (and not at end of lab sessions) to avoid any potential influence that our power manipulation might otherwise have on people’s self-report of PBC and PSOP. All participants (*n* = 135) completed this survey.

### Experimental Procedure: Power Manipulation and Assessment of Interoceptive Accuracy

Participants came to the lab individually. They were led to believe that the experiment would entail working in teams with another participant, who presumably had not arrived yet (in reality, participants did not engage in any teamwork task). The experimenter asked participants whether they would be willing to assist in calibrating a set of physiological sensors until their partner arrived–a request all agreed to. The experimenter then attached physiological sensors to participants’ non-dominant hands and instructed them to refrain from making any hand or body movements.

While participants were alone in the room, their baseline physiological measures, including blood volume pulse and skin conductance response (SCR) were recorded during an interval of 2 min (serving as a baseline measure). Throughout the experiment, heartbeat signals were acquired using a pulse transducer attached to the participant’s third finger and GSR signals were acquired using finger electrodes attached to the participant’s second (index) and fourth fingers. The sensors were connected to a physiological data acquisition unit (PowerLab 8/35, AD Instruments), sampling at 1 kHz, which transferred recorded signals to a PC running LabChart Pro software (AD Instruments), which derived physiological measures.

Next, we manipulated power using a “manager-subordinate” role-playing task, a well-established and widely used procedure to induce feelings of having or lacking power among participants (Guinote et al., 2002; Galinsky et al., 2003, 2008; Guinote, 2007; Overbeck and Droutman, 2013). As part of this task, participants completed a bogus questionnaire, ostensibly designed to identify their role (e.g., manager, subordinate, or colleague) in the upcoming teamwork task. In reality, we used random assignment to assign participants to three experimental conditions: *high power, low power*, and *control*. However, administering this questionnaire was essential to make participants believe that their assigned roles were legitimate. After completing the (bogus) questionnaire, participants were randomly assigned to a high-power (i.e., manager role), low-power (i.e., subordinate role), or control (i.e., colleague role) condition and received a description of their role.

More specifically, participants in the *high-power* experimental condition learned that they would be paired with another participant who would be their “subordinate” and that their task was to evaluate and judge their subordinate’s performance in a problem-solving task assigned to them. Additionally, they learned that they would determine which proportion of a designated monetary reward their “subordinate” would receive upon completing the task. In contrast, participants in the *low-power* experimental condition (i.e., subordinates) were informed that they would be paired with a “manager,” who would supervise and evaluate their performance in a problem-solving task, and that their “manager” had complete control over their monetary reward associated with the task. Finally, participants in the control condition, labeled as “colleagues,” were told that they would be matched with another “colleague” to complete a problem-solving task, and that they both would receive a designated monetary reward after completing the task. Accordingly, and in line with the definition of social power (Keltner et al., 2003; Fiske, 2010), the powerful (i.e., managers) had more control over the financial resources, could determine the outcomes of the powerless (i.e., subordinates), and were less dependent on them. Conversely, the powerless had less control over financial resources and their outcomes were more dependent on the powerful. Lastly, people in the control condition (i.e., colleagues) had no specific influence on each other’s outcomes (see the Supplementary Material for detailed instructions used for power manipulation).

Following the power manipulation, and while participants were waiting for their team member to supposedly arrive for the teamwork task, physiological measures were recorded again for another interval of 2 min. We conducted this second measurement to compare the results with participants’ baseline physiological responses, recorded before the power manipulation. Therefore, we could examine the impact of the power manipulation on participants’ physiological responses, and assess the robustness of our expected results by controlling for changes in physiological responses.

Subsequently, interoceptive accuracy was measured using the Schandry heartbeat-detection task (Schandry, 1981). Specifically, participants counted their felt heartbeats in two sets of three randomly ordered trials of varying duration (25, 35, and 45 s) marked by audio-visual start and stop cues. After each trial, participants typed the number of heartbeats they had felt during that interval. No performance feedback was provided. Throughout the task, participants were not permitted to take their pulse, or to use any other physical strategy such as holding their breath that could facilitate detection of heartbeats.

To ensure that participants’ performance in the Schandry task resulted from genuine interoceptive ability rather than from inferred heartbeats based on counting time, we also administered a time-estimation control task (Ehlers and Breuer, 1992). Similar to Dunn et al. (2010), three time-estimation trials were embedded within the six heartbeat-detection trials. After the first three heartbeat-detection trials, participants estimated the duration of three randomly ordered time intervals varying in length (23, 40, and 56 s), instead of counting their heartbeats, followed again by three heartbeat-detection trials.

Last, physiological sensors were detached from participants who were then guided to a separate room where they completed a series of self-report measures. Particularly, the survey consisted of two manipulation-check questions, followed by the PANAS scale (Watson et al., 1988) to assess participants’ affective state. Additionally, in this survey, we also measured variables that, though not critical to our main hypothesis, may possibly influence interoceptive accuracy, as suggested by past findings. We aimed to examine the robustness of our main findings by controlling for potential effects of these additional variables. Specifically, participants indicated the following: a subjective estimate of their resting/normal heart rate (in beats per minute), frequency of physical exercise, records of heart conditions, degree of task involvement during the experiment, gender, age, height, and weight. Height and weight were used to calculate participants’ BMI. We have included analyses of our data using these additional self-reported measures in the Supplementary Material of this paper. After completing the survey, participants were debriefed, thanked, and paid.

## Results

### Data Inspection and Exclusion

All participants completed the experiment. However, the data from four participants were excluded prior to the analysis because artifacts observed in their heart trace rendered uncertainty about the number of recorded heartbeats. Accordingly, our final sample for the main and follow-up analyses consisted of 131 participants (*n*_powerless_ = 44, *n*_control_ = 42, *n*_powerful_ = 45). As summarized in **Table 1**, we did not find any significant differences between experimental conditions with respect to participants’ age, BMI, and gender composition, indicating a homogenous distribution of participants across experimental conditions, resulting from random assignment^1^.

**Table 1:** Sample characteristics across experimental conditions.

	Powerless (*n* = 44)	Control (*n* = 42)	Powerful (*n* = 45)	Test statistic	*p*-value
Gender composition (Female/Male)	30/14	22/20	34/11	χ^2^(2, *N* = 131) = 5.36	0.07
BMI	22.13 (3.65) [21.16, 23.11]	22.68 (3.55) [21.68, 23.67]	22.07 (2.48) [21.11, 23.03]	*F*(2,128) = 0.45	0.64
Age	23.25 (4.59) [22.04, 24.46]	24.07 (4.05) [22.83, 25.31]	23.75 (3.48) [22.55, 24.95]	*F*(2,128) = 0.44	0.64

*Numbers in parentheses represent the standard deviations (SD); numbers in square brackets represent 95% confidence intervals for the mean estimates.*

### Power Manipulation Check

Participants indicated the extent to which they perceived (*a*) themselves, and (*b*) their team members in charge and control of the outcomes in the upcoming teamwork task, using 7-point scales with 1 anchored as “not at all in control” and 7 as “very much in control.” A 3 (power condition: high-power vs. low-power vs. control) × 2 (perceived control: self vs. team member) ANOVA with repeated measures on the second factor was conducted. Results revealed only a significant interaction between the two factors, *F*(2,128) = 17.96, *p* < 0.001, η^2^ = 0.30.

As expected, participants in the high-power experimental condition perceived themselves (*M*_self_ = 4.56, *SD* = 1.37), but not their team members (i.e., subordinates) (*M*_team member_ = 3.21, *SD* = 1.64, *F*(1,128) = 20.50, *p* < 0.001, 95% CI_Mean-Difference_ [0.76, 1.94], *d* = 0.82), to be *more* in charge and control of the outcomes. Conversely, participants in the low-power condition perceived themselves (*M*_self_ = 4.14, *SD* = 1.75), but not their team members (i.e., the managers) (*M*_team member_ = 5.32, *SD* = 1.54, *F*(1,128) = 15.33, *p* < 0.001, 95% CI_Mean-Difference_ [0.59, 1.78], *d* = 0.72), to be *less* in control of the outcomes. Finally, participants in the control condition (i.e., colleagues), perceived themselves (*M*_self_ = 4.17, *SD* = 1.41) and their team member (*M*_team member_ = 3.87, *SD* = 1.37, *F* < 1, *p* = 0.33, 95% CI_Mean-Difference_ [-0.31, 0.92]) to be equally in control of the outcomes. These results indicate that our power manipulation was successful. We therefore proceed to test our main proposition that the powerful, relative to the powerless and people in the control condition, should be more accurate in detecting their bodily signals (i.e., heartbeats).

### The Effect of Power on Interoceptive Accuracy

For each participant, interoceptive accuracy (IA) was calculated as the mean score of the heartbeat-detection performance across the six trials using the following transformation (Pollatos et al., 2007b):

$$\text{IA}=\frac{1}{6}\sum \left(1-\frac{\left|\text{Recorded heartbeats-Counted heartbeats}\right|}{\text{Recorded heartbeats}}\right)\times 100.$$

This transformation creates a percentage score varying between 0 and 100 for each person, with higher scores indicating a smaller difference between recorded and counted heartbeats, and thus higher interoceptive accuracy. A one-way ANOVA revealed a significant effect of power conditions on interoceptive accuracy, *F*(2,128) = 5.76, *p* = 0.004, η^2^ = 0.08. Planned contrasts showed that participants in the high-power experimental condition (*M* = 65.79, *SD* = 14.18) demonstrated greater interoceptive accuracy than did those in the control (*M* = 53.48, *SD* = 24.60, *F*(1,128) = 6.93, *p* = 0.01, 95% CI_Mean-Difference_ [3.05, 21.57], *d* = 0.61) and low-power conditions (*M* = 51.20, *SD* = 25.14, *F*(1,128) = 9.96, *p* = 0.002, 95% CI_Mean-Difference_ [5.44, 23.74], *d* = 0.71). There was no significant difference in interoceptive accuracy between the control and low-power conditions (*F* < 1, *p* = 0.63, 95% CI _Mean-Difference_ [-7.03, 11.59]). These findings corroborate our hypothesis that, relative to the powerless and people in the control condition, the powerful are more accurate in perceiving their bodily signals. We reasoned that this greater accuracy was the result of inward attentional shift caused by experiencing power. However, our findings may be explained by processes other than an inward attentional shift, through which power might have influenced interoceptive accuracy. In the following, we examine and rule out those alternative processes.

### Addressing Alternative Processes

#### Arousal

Our power manipulation might have increased participants’ level of physiological arousal, which can facilitate access to bodily signals and result in superior performance in the Schandry heartbeat-detection task (Pollatos et al., 2007a). To test this explanation, we analyzed participants’ physiological arousal as a function of their power condition using three different markers of arousal. As explained in the experimental procedure, we measured participants’ physiological markers before (i.e., the baseline) and after power manipulation. Because participants were randomly assigned to experimental conditions and the baseline physiological measures were recorded before power was manipulated, we did not expect differences in baselines measures between experimental conditions. Confirming this, separate ANOVAs revealed that neither participants’ baseline heart rates (HR), *F*(2,128) = 0.47, *p* = 0.63, nor their baseline heart-rate variability (RMSSD) were significantly different across power conditions, *F*(2,128) = 0.26, *p* = 0.77. Similarly, participant’s baseline SCR did not significantly differ across power conditions, *F*(2,128) = 0.34, *p* = 0.71.

Having confirmed that our experimental conditions did not differ in their baseline physiological measures, we continued to examine whether our power manipulation caused changes in participants’ arousal, which might then explain the link between power and interoceptive accuracy. For each participant, changes in heart rate (ΔHR), heart-rate variability (ΔRMSSD), and skin conductance response (ΔSCR) were calculated using their physiological responses recorded before (i.e., baseline) and after the power manipulation. Separate ANOVAs revealed that our power manipulation did not change participants’ heart rate (ΔHR, *F* < 1, *p* = 0.84) or their heart-rate variability (ΔRMSSD, *F* < 1, *p* = 0.69). However, our power manipulation had a significant effect on participants’ ΔSCR, *F*(2,128) = 23.24, *p* < 0.001, η^2^ = 0.27, such that participants in the low-power experimental condition showed a larger SCR change (*M* = 0.23, *SD* = 0.16) than did participants in the control (*M* = 0.09, *SD* = 0.08, *F*(1,128) = 33.09, *p* < 0.001, 95% CI_Mean-Difference_ [0.09, 0.19], *d* = 1.11), and high-power conditions (*M* = 0.09, *SD* = 0.08, *F*(1,128) = 36.40, *p* < 0.001, 95% CI_Mean-Difference_ [0.10, 0.19], *d* = 1.11). Interestingly, the change in SCR was not significantly different between participants in the high-power and control conditions (*F* < 1, *p* = 0.86), suggesting that it is unlikely that the power holders’ physiological arousal accounts for their greater interoceptive accuracy.

Consequently, across three measures of physiological arousal, results of our analyses are inconsistent with the idea that experiencing power renders greater interoceptive accuracy through the arousal path. Nevertheless, to ensure the robustness of the relation between power and interoceptive accuracy, we further conducted a series of regression analyses^2^ in which we statistically controlled for the effects of physiological markers of arousal in our main analysis. When controlling for changes in heart rate (ΔHR) as a covariate, we found a marginally significant effect of ΔHR on interoceptive accuracy, (*b* = 1.29, *SE_b_* = 0.68, *t*(127) = 1.89, *p* = 0.06, 95% CI*_b_* [-0.06, 2.65]), indicating that irrespective of their experimental power condition, participants with a larger change in HR detected their heartbeats more accurately. Importantly, however, the effect of power on interoceptive accuracy remained significant, such that participants in the high-power experimental condition detected their heartbeats more accurately than did those in the control (*b*_Powerful vs. Control_ = 12.54, *SE_b_* = 4.63, *t*(127) = 2.71, *p* = 0.008, 95% CI*_b_* [3.37, 21.71]), and low-power conditions (*b*_Powerful vs. Powerless_ = 14.37, *SE_b_* = 4.58, *t*(127) = 3.14, *p* = 0.002, 95% CI*_b_* [5.31, 23.43]). Therefore, controlling for the effect of ΔHR did not change the pattern or significance of our main findings.

Next, when controlling for changes in heart-rate variability (ΔRMSSD), ΔRMSSD did not significantly predict interoceptive accuracy (*t* < 1, *p* = 0.58), while the effect of power on interoceptive accuracy remained significant, consistent with our proposition (*b*_Powerful vs. Control_ = 12.21, *SE_b_* = 4.69, *t*(127) = 2.60, *p* = 0.01, 95% CI*_b_* [2.92, 21.50]; *b*_Powerful vs. Powerless_ = 14.70, *SE_b_* = 4.64, *t*(127) = 3.17, *p* = 0.002, 95% CI*_b_* [5.52, 23.87]). Similarly, when controlling for changes in skin conductance responses (ΔSCR) as a covariate in our main analysis, ΔSCR did not significantly predict interoceptive accuracy (*t* < 1, *p* = 0.97); however, the effect of power on interoceptive accuracy remained significant (*b*_Powerful vs. Control_ = 12.31, *SE_b_* = 4.70, *t*(127) = 2.62, *p* = 0.01, 95% CI*_b_* [3.01, 21.60]; *b*_Powerful vs. Powerless_ = 14.51, *SE_b_* = 5.26, *t*(127) = 2.76, *p* = 0.007, 95% CI*_b_* [4.10, 24.92]).

To sum, these analyses indicate that compared to people in the powerless and control conditions, having power does not increase participants’ arousal level. Therefore, physiological arousal is unlikely to be the mechanism through which power affects interoceptive accuracy. Moreover, we found that the effect of power on interoceptive accuracy is robust and remains significant, even after controlling for participants’ physiological arousal^3^ (see **Table 2** for a descriptive summary of arousal markers as a function of experimental power conditions).

**Table 2:** Physiological and self-reported measures as a function of social power.

	Powerless (*n* = 44)	Control (*n* = 42)	Powerful (*n* = 45)
Interoceptive accuracy (IA)	51.20 (25.14) [44.69, 57.70]	53.48 (24.60) [46.82, 60.13]	65.79 (14.18) [59.36, 72.22]
ΔHR^†^	-0.83 (3.5) [-1.66, 0.001]	-0.48 (2.29) [-1.33, 0.37]	-0.66 (2.40) [-1.48, 0.17]
ΔRMSSD^†^	-0.04 (0.58) [-0.17, 0.08]	-0.12 (0.38) [-0.25, 0.004]	-0.09 (0.20) [-0.21, 0.04]
ΔSCR^†^	0.23 (0.16) [0.20, 0.27]	0.09 (0.08) [0.06, 0.13]	0.09 (0.08) [0.05, 0.12]
Self-reported positive affect	3.18 (0.63) [2.99, 3.37]	3.18 (0.63) [2.99, 3.37]	3.10 (0.63) [2.92, 3.29]
Self-reported negative affect	1.75 (0.56) [1.60, 1.90]	1.60 (0.44) [1.45, 1.75]	1.62 (0.49) [1.47, 1.77]
Time-estimation accuracy	76.86 (14.50) [71.52, 82.20]	74.02 (18.88) [68.55, 79.49]	72.25 (19.88) [66.97, 77.53]

*Numbers in parentheses represent the standard deviations (SD); numbers in square brackets represent 95% confidence intervals for the mean estimates. ^†^The change scores (Δ) were calculated by subtracting participants’ pre-manipulation physiological responses (i.e., baselines) from their post-manipulation physiological responses.*

#### Affect

Positive affect has been found to increase self-focused attention (Silvia and Abele, 2002), which can enhance interoceptive accuracy (Ainley et al., 2012). Since an elevated feeling of power has been proposed to increase positive affect (Keltner et al., 2003), our findings might be explained by participants’ affective states, rather than by their interoceptive attention shift. To test this explanation, we examined the effect of power on participants’ self-reported affective states, measured after power manipulation, using the PANAS (Watson et al., 1988). A series of one-way ANOVAs demonstrated that our power manipulation did not influence participants’ positive, *F*(2,128) = 0.21, *p* = 0.81, or negative affect, *F*(2,128) = 1.13, *p* = 0.33. Therefore, participants’ affective state is unlikely to be the underlying mechanism for our effect.

Additionally, to test the robustness of our main findings, we included participants’ affective states as covariates in the main analysis. Results of a regression analysis revealed that neither participants’ positive affect (*t* < 1, *p* = 0.71), nor their negative affect (*t* < 1, *p* = 0.40) significantly predicted their interoceptive accuracy. Importantly, controlling for participants’ affective states did not change the significance or pattern of our main findings. That is, participants in the high-power experimental condition were more accurate in detecting their interoceptive signals than were those in the control (*b*_Powerful vs. Control_ = 12.17, *SE_b_* = 4.71, *t*(126) = 2.58, *p* = 0.01, 95% CI*_b_* [2.85, 21.48]), and low-power conditions (*b*_Powerful vs. Powerless_ = 14.93, *SE_b_* = 4.68, *t*(126) = 3.19, *p* = 0.002, 95% CI*_b_* [5.68, 24.18]).

Overall, these analyses indicate that our power manipulation did not influence either positive or negative affective states, and that participants’ affective state could not account for their interoceptive accuracy (see **Table 2** for a descriptive summary of affective states as a function of experimental power conditions).

#### Goal Pursuit

Past research has demonstrated that having power increases goal-directed behavior (Guinote, 2007). Relating to our experiment, one intriguing possibility could be that our experimental setting might have activated “intentions for accuracy” among participants. Consequently, the better pursuit of this goal (i.e., trying to be accurate in the assigned tasks) by the powerful, rather than an inward attentional shift caused by experiencing power, might be the underlying driver of our effect. To rule out this possibility, we examined participants’ accuracy in the time-estimation task, embedded within the heartbeat-detection trials. We reasoned that if powerholders’ greater interoceptive accuracy was merely the result of their superior pursuit of a goal for accuracy, then the powerful should also perform more accurately in the time-estimation task.

An average accuracy score across the three time-estimation trials was calculated for each participant. A one-way ANOVA revealed that participants’ experimental condition did not influence their time-estimation accuracy *F*(2,128) = 0.75, *p* = 0.47, indicating that goal pursuit is not a plausible explanation for our findings (see **Table 2** for the summary of time-estimation accuracy as a function of experimental power conditions).

Finally, to test the robustness of the relation between power and interoceptive accuracy, we included participants’ accuracy in time estimation as a covariate in the main analysis. Results of a regression analysis revealed a positive and significant main effect of accuracy in time estimation (*b* = 0.28, *SE_b_* = 0.11, *t*(127) = 2.65, *p* = 0.009, 95% CI*_b_* [0.07, 0.49]), indicating that, irrespective of their experimental power conditions, participants who were more accurate in counting time were also more accurate in detecting their heartbeats. Notably, however, controlling for participants’ accuracy in counting time did not influence the pattern or significance of our main findings. Participants in the high-power experimental condition were more accurate in detecting their heartbeats than were those in the control (*b*_Powerful vs. Control_ = 12.80, *SE_b_* = 4.58, *t*(127) = 2.80, *p* = 0.006, 95% CI*_b_* [3.75, 21.86]), and low-power conditions (*b*_Powerful vs. Powerless_ = 15.88, *SE_b_* = 4.54, *t*(127) = 3.49, *p* = 0.001, 95% CI*_b_* [6.89, 24.87]).

To conclude, results of our analyses using both physiological and self-reported data are inconsistent with explanations that would attribute the process underlying the relation between power and interoceptive accuracy to effects other than powerholders’ inward attentional shift. Particularly, we demonstrated that the effect of power on interoceptive accuracy cannot be explained through participants’ physiological arousal, affective state, or intention for accuracy. In the following section, we use participants’ dispositional characteristics, measured two weeks before the experiment, to shed further light on the process underlying our effect and to generalize our findings beyond experimental power manipulations in the laboratory.

### The Role of Dispositional Characteristics

#### Body Consciousness

We argued that the powerful are more accurate in perceiving their bodily signals, mainly because having power shifts attentional resources inward. Consistent with this argument, one should expect that the effect of power on interoceptive accuracy would be stronger (weaker) among individuals with a lower (higher) chronic tendency to attend to their internal sensations. In other words, among people with a *higher* chronic tendency to attend to internal sensations, experiencing power should not render further accuracy in perceiving bodily signals (presumably due to a ceiling effect). However, if having power indeed shifts people’s attentional resources inward, it should have a bigger impact on interoceptive accuracy among those with a *lower* chronic tendency to attend to bodily signals. To test this hypothesis, we used individuals’ differences in attention to internal sensations, measured two weeks before the experiment, using the private body consciousness subscale (PBC) of the Body Consciousness Questionnaire (Miller et al., 1981). The PBC is one of the three subscales of the Body Consciousness Questionnaire and measures how sensitive individuals are in attending to their internal and bodily sensations (e.g., “I am attentive to internal bodily tensions”). The other two subscales are Public Body Consciousness and Body Competence, which we did not measure in our survey, as they were irrelevant to our hypothesis.

A general linear model (GLM) procedure was used, with power, PBC (mean-centered), and their interaction term as independent variables, and participants’ interoceptive accuracy as dependent variable. Results revealed a significant main effect of power condition *F*(2,125) = 5.97, *p* = 0.003, η^2^ = 0.08, but not of PBC *F*(1,125) = 1.17, *p* = 0.28 on interoceptive accuracy. More importantly, and as expected, the interaction between power and PBC was significant *F*(2,125) = 3.34, *p* = 0.039, η^2^ = 0.05. To specify the precise pattern of the power × PBC interaction, we examined the effects of power conditions (dummy coded to compare the high-power condition with control and low-power conditions) at one standard deviation above (high PBC) and below (low PBC) the mean of PBC, using a series of regressions (Aiken and West, 1991).

As expected, results of this analysis revealed that among high-PBC participants, the powerful did not show more interoceptive accuracy, than did those in the control (*b*_Powerful vs. Control_ = 7.74, *SE_b_* = 7.05, *t*(125) = 1.10, *p* = 0.28, 95% CI*_b_* [-6.22, 21.70]) and low-power conditions (*b*_Powerful vs. Powerless_ = 3.39, *SE_b_* = 6.87, *t*(125) = 0.49, *p* = 0.62, 95% CI*_b_* [-10.20, 16.99]). However, consistent with our reasoning, among low-PBC participants, the powerful were significantly more accurate in perceiving their bodily signals, than were those in the control (*b*_Powerful vs. Control_ = 15.66, *SE_b_* = 6.39, *t*(125) = 2.45, *p* = 0.016, 95% CI*_b_* [3.00, 28.31]) and low-power conditions (*b*_Powerful vs. Powerless_ = 27.10, *SE_b_* = 6.45, *t*(125) = 4.20, *p* < 0.001, 95% CI*_b_* [14.34, 39.86]).

**FIGURE 1:**
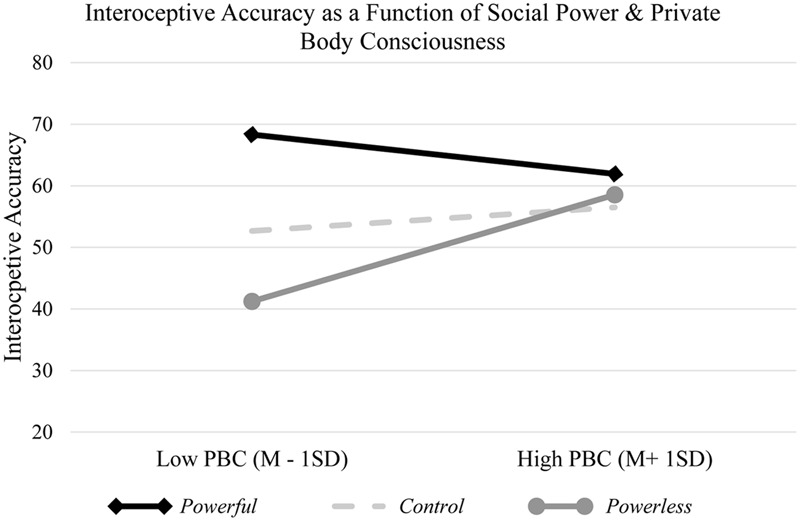
Interoceptive accuracy as a function of experimental conditions of power and participants’ PBC. The experience of having power does not further increase interoceptive accuracy among individuals with a higher chronic tendency to attend to bodily sensations (high PBC). However, relative to the powerless and participants in the control condition, having power significantly increases interoceptive accuracy, among individuals with a lower chronic tendency to attend to their bodily sensations (low PBC).

These findings underscore the role of inward attentional shift in the relation between power and interoceptive accuracy. Relative to people in the low-power and control conditions, the powerful perceive their somatic signals more accurately, and this effect is more pronounced among those with a lower chronic tendency to attend to internal sensations. However, among people whose attention was chronically turned inward to monitor bodily sensations (i.e., high PBC), experiencing power did not further increase accuracy in perceiving bodily signals^4^ (see **Figure 1**).

#### Personal Sense of Power (PSOP)

Individuals develop a general sense of their power over time and across social contexts (Anderson et al., 2012). A different approach to test our main hypothesis is to examine whether participants’ chronic sense of their power (PSOP) also predicts interoceptive accuracy, similar to how their situational experience of power does. To this end, we examined joint effects of situationally induced feelings of power, as manipulated in the lab, and participants’ dispositional sense of power (PSOP), which we measured two weeks before the experiment, on interoceptive accuracy.

A stepwise linear regression procedure was applied. First, we regressed participants’ interoceptive accuracy scores on power conditions (dummy coded to compare the high-power experimental condition with the control and low-power conditions) and PSOP (mean-centered). As expected, results revealed that a situational experience of power significantly increased people’s interoceptive accuracy compared to that of their counterparts in the control (*b*_Powerful vs. Control_ = 11.52, *SE_b_* = 4.60, *t*(127) = 2.51, *p* = 0.013, 95% CI*_b_* [2.43, 20.62]) and low-power conditions (*b*_Powerful vs. Powerless_ = 13.06, *SE_b_* = 4.57, *t*(127) = 2.86, *p* = 0.005, 95% CI*_b_* [4.01, 22.11]). Moreover, people’s chronic sense of power (PSOP) was also positively and significantly associated with their interoceptive accuracy (*b* = 5.85, *SE_b_* = 2.35, *t*(127) = 2.49, *p* = 0.014, 95% CI*_b_* [1.20, 10.50]), indicating that, independent of their experimental condition, those with higher dispositional sense of power were more accurate in perceiving their bodily signals. Next, we entered the respective interaction terms between power conditions (dummy coded) and PSOP (mean-centered) into the model. Results revealed that interaction terms were not significantly predicting interoceptive accuracy (*p*s > 0.16) and that including them in the analysis did not significantly increase the first model’s explanatory power, Δ*R*^2^ = 0.01, *F*_change_ (2,125) = 0.99, *p* = 0.37. Therefore, these analyses demonstrate that situationally induced feelings of power as well as dispositional sense of power have similar and independent effects on interoceptive accuracy. The observed independence between experimentally induced feeling of power and dispositional sense of power in our findings are consistent with recent research showing that one’s positional power (e.g., one’s role as a manager) and sense of power have similar but independent effects on various state measures in daily life (Smith and Hofmann, 2016). Consequently, these results generalize our proposition by showing that social power renders more accurate perception of bodily signals, whether it stems from situational or chronic sources.

## Discussion and Future Directions

Attention is fundamental in binding humans (and other primates) into social organizations and promotes safety and access to resources (Chance and Jolly, 1970; Fiske, 2010). However, attention is a limited resource, and interoceptive and exteroceptive stimuli compete for organisms’ limited information processing capacity (Pennebaker and Lightner, 1980; Pennebaker, 1982). Consequently, factors that shift attention inward should promote higher access to bodily signals and enhance interoceptive accuracy (Gibbons et al., 1979; Scheier et al., 1979; Ehlers and Breuer, 1996; Carver, 2011). In the present research, we proposed and provided experimental evidence that social power is one such a factor.

Building on research showing that social power liberates the self from external influences and increases self-focused attention, we proposed that experiencing power leads to more accurate perception of bodily signals. Consistent with our proposition, after power was manipulated, people in the high-power experimental condition outperformed those in control and low-power conditions in the Schandry heartbeat-detection task. We demonstrated that the effect of power on interoceptive accuracy was not explained by participants’ physiological arousal, affective state, or general intention for accuracy. Rather, the effect of situationally induced feelings of power on interoceptive accuracy was dependent on individuals’ chronic tendency to focus on their visceral sensations, signifying the role of inward attentional shift with respect to our hypothesis. Finally, we demonstrated that chronic sources of feeling powerful also predicted people’s interoceptive accuracy similar to, and independent of, how their situationally induced feeling of power did. While in this manuscript, we limited our analyses to variables that were central to our main hypothesis, further analyses of our data using other variables that we collected at the end of our experiment (e.g., gender, BMI, participants’ exercise frequency, etc.) can be found in the Supplementary Material accompanying this article.

Experiencing power has been found to increase approach-related processes (Keltner et al., 2003) such as action tendencies and risk-taking (Galinsky et al., 2003; Anderson and Galinsky, 2006), and to facilitate focus on desired goals and outcomes (Slabu and Guinote, 2010; Hirsh et al., 2011). Our research contributes to these findings by suggesting a novel pathway, namely increased interoceptive accuracy, through which power and hierarchy can influence decision-making and behavior, thus providing several avenues for future research.

First, sensitivity to cardiac signals has been shown to involve brain structures associated with monitoring and perceiving emotions and visceral states (Craig, 2004, 2011; Critchley et al., 2004). Accordingly, our findings suggest that having power should intensify the experience of feelings and visceral states, which are known to markedly influence judgements and behavior (Loewenstein, 1996; Schwarz and Clore, 2007). We thus predict that identical visceral states (e.g., hunger, thirst, and sexual desire), and sources of affective experience, have a greater impact on the powerful than on the powerless. For instance, research has shown that people with activated sexual appetite (i.e., experiencing a hot, visceral state) are more likely to opt for smaller-sooner monetary and non-monetary rewards, foregoing larger-later rewards (Wilson and Daly, 2004; Van den Bergh et al., 2008). Relevant to the present research, our findings suggest that the powerful, relative to the powerless and people in the control condition, should show increased reward-seeking intentions and behavior when experiencing a hot, visceral state (e.g., sexual desire).

Moving beyond reward-related tendencies in consumption domains, our findings also offer novel research possibilities on how power might shape social interactions, both in person perception and in reaction to other people’s (economic) offers. In relation to person perception, research has shown that the arousal experienced when interacting with others modulates one’s preference toward those people (Stephan et al., 1971). There is a significant overlap between brain areas associated with interoceptive accuracy and those related to experiencing arousal (Pollatos et al., 2007b,c). Given the link between power and interoception, we predict that power should intensify the experience of arousal during social interactions, which in turn should influence powerholders’ expectations from and behavior toward other people. This prediction is consistent with past findings showing that social power heightens expectations of sexual interest from subordinates of the opposite sex (Kunstman and Maner, 2010), and that it increases people’s infidelity among both powerful men and women (Lammers et al., 2011). Future research can fruitfully investigate the power-interoception link to elucidate the nature of some of the corruptive effects of power in social interactions.

Another interesting avenue for future research is to investigate the role of interoceptive accuracy in how social power modulates reactions to unfair offers. Power has been found to heighten sensitivity to unfair offers (Sawaoka et al., 2015). The tendency to reject unfair offers is modulated by emotional and physiological reactions mediated by interoceptive accuracy (Dunn et al., 2012). Our findings therefore suggest that increased perception of bodily signals among the powerful when receiving an unfair offer should mediate the relation between power and rejection of unfair offers, for instance in ultimatum games.

Future research can also investigate how hierarchical dynamics (Sapolsky, 2005) might modulate our current findings. For example, when the power position is stable, we predict that the powerful are more accurate in perceiving bodily signals, than are the powerless and people in the control condition. Conversely, when there is fierce competition for valued resources (e.g., food, money, and mating) within the hierarchy and thus the power position is unstable, having power should not cause superior interoceptive accuracy. This is because safeguarding one’s power in an unstable hierarchy demands shifting attentional resources outward to predict and monitor threats, decreasing accuracy in perceiving interoceptive signals. Similarly, another feature of social power that might influence its effect on interoceptive accuracy is legitimacy. When power is illegitimate, behavioral markers of having power, such as action tendencies and risk-taking, are drastically reduced (Lammers et al., 2008), presumably due to enhanced stress and vigilance experienced by powerholders in that context. The stress for having an illegitimate power position should shift attentional resources outward for constantly monitoring potential threats. Therefore, we predict that illegitimacy should reduce the effect of power on accurate perception of bodily signals.

Furthermore, future research would benefit from examining whether other psychosocial resources that are correlated with power could engender similar effects on introspective accuracy. For instance, people’s social class might also predict their interoceptive accuracy. Upper social class is associated with increased social distance (Kraus and Keltner, 2009), lower stress (Adler et al., 1994; Adler and Ostrove, 1999; Sherman et al., 2012), and higher self-esteem (Twenge and Campbell, 2002). A recent conceptualization of social class (Kraus et al., 2012) posits that belonging to the upper social class increases individualistic orientation and promotes reliance on one’s internal states, emotions, and goals. These effects are comparable to those of social power, as described in this research, and thus suggest that social class could also predict people’s interoceptive accuracy, similar to how power does.

Additionally, research on the feeling of powerlessness can also benefit from our findings. If increased interoceptive accuracy is an important pathway through which social power affects decision-making and behavior, then situational induction of factors known to increase interoceptive accuracy should yield similar effects among the powerless. For instance, exposure to one’s mirror image, a well-known method to increase self-focused attention and interoceptive accuracy (Ainley et al., 2012), should increase reliance on affective and visceral states among the powerless, a tendency that exists among the powerful by default.

Finally, our research also contributes to the literature on interoceptive accuracy by highlighting the state-dependent nature of interoceptive accuracy. Particularly, we showed that a brief experience of power in a social situation suffices to enhance the perception of bodily signals. Therefore, we contribute to a recent and growing body of research aiming at identifying situational factors that modulate interoceptive accuracy. For instance, situational exposure to one’s mirror image (Ainley et al., 2012), and focusing on narrative aspects of the self (Ainley et al., 2013) have been also found to increase interoceptive accuracy. Future research would benefit from investigating other situational and context-sensitive factors that might increase or decrease interoceptive accuracy.

## Conclusion

Our findings offer a novel account for understanding and explaining the effects of social power. The present findings suggest that the experience of having power informs powerholders of their visceral signal by promoting a state of enhanced interoceptive accuracy. Therefore, power is not only a psychosocial resource that buffers external influences, but it is also a gateway to one’s feelings and visceral drives. The intensified experience of inner sensations subsequently shapes powerholders’ decisions and behaviors. As the opening quote of this paper implies, relying on one’s gut feelings may be inevitable in the world of the powerful.

## Ethics Statement

All participants gave their written informed consent in accordance with the Declaration of Helsinki, before participating in the experiment and their data were treated anonymously. This research was conducted according to the guidelines provided by the Norwegian Social Science Data Services (Norsk Samfunnsvitenskapelig Datatjeneste), the national authority in research ethics in Norway. Additionally, the research ethics committee at BI Norwegian Business School approved this research and its experimental procedure, prior to data collection.

## Author Contributions

All co-authors contributed significantly at different stages of this research. In particular, MM-J, LdM, and LW developed the initial research idea. MM-J, KK, LdM, and LW designed the experiment. MM-J, KK, LdM, and EG conducted the experiment and collected data. EG prepared the physiological data. MM-J and KK analyzed data. MM-J wrote the manuscript. KK, LdM, and LW revised the manuscript. This manuscript has not been published and is not under review at any other journal.

## Conflict of Interest Statement

The authors declare that the research was conducted in the absence of any commercial or financial relationships that could be construed as a potential conflict of interest.

## Footnotes

1 Past research provides inconsistent evidence as to whether gender, age and BMI influence interoceptive accuracy. While some findings suggest that these variables might interfere with interoception (Cameron, 2001; Herbert et al., 2013), other findings have found no evidence for such effects (Dunn et al., 2007, 2010). Nevertheless, these variables were measured in our study and we did not observe any significant differences across experimental conditions with respect to these variables. Moreover, the Supplementary Material accompanying this article includes further analysis to check the robustness of our main findings with respect to these variables.

2 In all the reported regression analyses throughout this article, continuous variables were mean centered and experimental conditions (high-power, control, and low-power) were dummy coded. Because our main hypothesis pertains to the experience of *“having”* power, we always considered the high-power condition as the reference group in our dummy coding. This coding enabled us to quantify and compare participants’ interoceptive accuracy in the high-power experimental condition with those of control and low-power conditions.

3 Observing larger ΔSCR among the low-power participants is consistent with past findings showing that lack of power is associated with greater stress and heightened vigilant state (Fiske, 2010; Smith and Hofmann, 2016). Importantly, however, as our data suggests, ΔSCR does not affect interoceptive accuracy of *the powerless*, relative to people in the control condition.

4 Although it is conventional to conduct spotlight analysis at one standard deviation above (i.e., high) and below (i.e., low) the mean of the continuous moderating variable (i.e., PBC), this approach is suboptimal in that these two points are chosen arbitrarily and thus do not specify the exact point in the range of the continuous moderating variable in which the difference between high-power condition and the other two experimental conditions became simultaneously significant. Therefore, following recommendations by Spiller et al. (2013), we conducted a floodlight analysis using Johnson and Neyman’s approach on the range of PBC (i.e., from 1 to 5). Results revealed that power significantly increased people’s interoceptive accuracy compared to that of their counterparts in the control (*b*_Powerful vs. Control_ = 10.21, *SE_b_* = 5.16, *t*(127) = 1.98, *p* = 0.05, 95% CI*_b_* [0.01, 20.42]) and low-power conditions (*b*_Powerful vs. Powerless_ = 10.80, *SE_b_* = 5.06, *t*(127) = 2.13, *p* = 0.035, 95% CI*_b_* [0.79, 20.80]), for any PBC value below 3.57, but not for any PBC value greater than 3.57.
