# Supplementary material for: Social Power Increases Interoceptive Accuracy
Source: Front Psychol. 2017 Aug 3;8:1322. doi: 10.3389/fpsyg.2017.01322 (PMC5541025; doi:10.3389/fpsyg.2017.01322)
Supplement: Supplementary file 1 [file Presentation_1.PDF]

# *Supplementary Material*

# Social Power Increases Interoceptive Accuracy

Mehrad Moeini-Jazani*[1], Klemens Knoferle[2], Laura de Molière[3], Elia Gatti[4], Luk Warlop[2]

[1]University of Groningen, Groningen, the Netherlands,

[2]BI Norwegian Business School, Oslo, Norway,

[3]University College London, London, United Kingdom,

[4]University of Sussex, Brighton, United Kingdom.

*** Correspondence:** Mehrad Moeini-Jazani, Faculty of Economics and Business, University of Groningen, Nettelbosje 2, 9747 AE Groningen, the Netherlands. E-mail: m.moeini.jazani@rug.nl

This document has two sections:

**S1.** Material for Power Manipulation Using the Role-Playing Task

**S2.** Further Data Analyses Using Self-Report Measures

## **S1. Material for Power Manipulation Using the Role-Playing Task**

We manipulated power using a "manager-subordinate" role-playing task, a well-established and widely used procedure to reliably induce feelings of having or lacking power among participants (Galinsky, Gruenfeld, & Magee, 2003; Galinsky et al., 2008; Guinote, 2007; Guinote, Judd, & Brauer, 2002; Overbeck & Droutman, 2013).

Participants in the *high-power* experimental condition read the following feedback on the screen:

> According to answers you provided to our online questionnaire, it seems that you have a "managerial" creativity style. This means that you are good at organizing tasks, instructing and evaluating team members' performance. Therefore, you are assigned the "manager" role for the subsequent teamwork task. You will be paired with someone having the "problem-solving" skill set, who will be your subordinate.
>
> As manager, your task is to evaluate your subordinate's performance in solving several problems assigned to your team. You will decide how to structure the process and the standards by which the subordinate's work will be evaluated. You should immerse yourself completely into your assigned role and make this experience as realistic as possible. Upon concluding the task, you will judge your subordinate's performance by completing a private evaluation questionnaire, which you will give to the experimenter. Your subordinate will not be able to see your evaluation.
>
> The amount of money your subordinate will receive depends on your evaluation of his/her performance in the task. After your evaluation, you will determine which proportion of a designated monetary reward your subordinate receives upon completing the task. Your subordinate will not have any opportunity to evaluate you or to influence your income. As manager, you will receive the full designated monetary reward, considered for this task.

Participants in the *low-power* experimental condition read the following:

> According to answers you provided to our online questionnaire, it seems that you have a "problem-solving" creativity style. This means that you are good at addressing problems and devising solutions. Therefore, you are assigned the "subordinate" role for the subsequent teamwork task. You will be paired with someone having the "managerial" skill set, who will be your manager.
>
> As a subordinate, your task is to generate creative solutions for several problems assigned to your team. Your performance in solving them will be evaluated by your manager. You should immerse yourself completely in your assigned role and make this experience as realistic as possible. Upon concluding the task, your manager will judge your creativity and performance in solving the problems by completing a private questionnaire, which he will give to the experimenter. You will not be able to see his/her evaluation.
>
> The amount of money you will receive for this task depends on your manager's evaluation of your performance. After evaluating your performance, your manager will determine which proportion of a designated monetary reward you will receive upon completing the task. You will not have any opportunity to evaluate your

manager or to influence his/her income. Your manager will receive the full designated monetary reward considered for this task.

Finally, participants in the *control* condition read the following:

According to the answers you provided to our online questionnaire, it seems that you have a “collegial” creativity style, which means that you are able to work with others in a team as well as individually. Therefore, you are assigned the “colleague” role for the subsequent teamwork task. You will be paired with a partner who is a “colleague,” like you.

As a colleague, your task is to solve several problems assigned to you. You should immerse yourself completely in your assigned role and make this experience as realistic as possible. You will receive the designated monetary reward considered for this task, after completing the task.

## S2. Further Data Analyses Using Self-Report Measures

***Role of Subjective Knowledge of Normal Heart Rate.*** Research has found that people with a more accurate knowledge/belief about their normal (i.e., resting) heart rate, are more accurate in the Schandry heartbeat-detection task (Brener, Knapp, & Ring, 1995). A strong test for the robustness of our hypothesis would be to see if the effect of power on interoceptive accuracy remains significant, after controlling for people’s knowledge accuracy regarding their resting heart rates. To that end, we asked participants to provide an estimate of their normal heart rate (i.e., beats per minute) at the end of the experiment. We then calculated participants’ knowledge accuracy about their normal/resting heart rates using the following formula:

Accuracy of knowledge/belief about resting HR =

$$\left(1 - \frac{|Recorded\ \ baseline\ heartbeats - Estimated\ resting\ heartbeats\ |}{Recorded\ \ baseline\ heartbeats}\right) \times 100$$

As expected, a one-way ANOVA revealed that our power manipulation did not significantly predict participants' knowledge accuracy of their heart rates ($F < 1$, $p = .91$). Next, we tested the robustness of our main results by statistically controlling for the effect of participants' knowledge accuracy about their resting heart rates, using a stepwise linear regression procedure. First, we regressed participants' interoceptive accuracy scores on power conditions (dummy coded to compare high-power condition with control and low-power conditions), and HR knowledge accuracy (mean-centered). Results of a regression analysis revealed a positive and significant main effect of HR knowledge accuracy ($b = 0.21$, $SE_b = 0.09$, $t(127) = 2.40$, $p = .02$, 95% $CI_b$ [0.04, 0.39]), indicating that independent of their experimental conditions, participants with a more accurate knowledge about their resting heart rates were also more accurate in the Schandry heartbeat detection task. Notably, however, results also revealed a significant main effect of power condition consistent with our main findings. Participants in the high-power experimental condition were more accurate in detecting their heartbeats than were those in the control ($b_{Powerful\ vs.\ Control} = 12.28$, $SE_b = 4.59$, $t(127) = 2.67$, $p = .009$, 95% $CI_b$ [3.19, 21.37]), and low-power conditions ($b_{Powerful\ vs.\ Powerless} = 14.21$, $SE_b = 4.54$, $t(127) = 3.13$, $p = .002$, 95% $CI_b$ [5.23, 23.20]).

Next, we added the respective interaction terms between Knowledge Accuracy about HR (mean-centered) and Power conditions (dummy coded). However, results of this analysis revealed that interaction terms were not statistically significant and adding those terms did not significantly increase the explanatory power of the first model $\Delta R^2 = .028$, $F_{change}(2,125) = 2.06$, $p = .13$. Therefore, the most accurate way to interpret the results is by considering the main effects as reported above.

***Role of BMI.*** We calculated participants' body mass index using their height and weight self-reports ($BMI = \frac{weight(kg)}{(height(m))^2}$). A stepwise linear regression procedure was applied to investigate the effect of power and BMI on interoceptive accuracy. First, we regressed participants' interoceptive

accuracy scores on power conditions (dummy coded to compare high-power condition with control and low-power conditions), and BMI (mean-centered). The effect of BMI on interoceptive accuracy was not statistically significant ($t < 1$, $p = .66$). However, as expected, the effect of social power on interoceptive accuracy remained significant and in line with our proposition ($b_{\text{Powerful vs. Control}} = 12.47$, $SE_b = 4.71$, $t(127) = 2.65$, $p = .009$, 95% $CI_b$ [3.15, 21.78]; $b_{\text{Powerful vs. Powerless}} = 14.60$, $SE_b = 4.64$, $t(127) = 3.15$, $p = .002$, 95% $CI_b$ [5.43, 23.78]). Next, when we entered the respective interaction terms between power conditions (dummy coded) and BMI (mean-centered) into the model, the interaction terms were not significantly predicting interoceptive accuracy (*ps*> .52). These analyses demonstrate that participants' BMI does not predict their interoceptive accuracy, nor does it moderate the relation between power and interoceptive accuracy.

***Role of Gender.*** We ran an ANOVA with participants' power (high power, control, low power) and gender (females = 86 vs. males = 45) as independent variables and interoceptive accuracy as a dependent variable. Results of this analysis revealed only a significant main effect of power $F(2,125) = 4.50$, $p = .01$, $\eta^2 = .07$. Neither the main effect of gender ($p = .16$), nor its interaction with power condition were significant ($p = .19$).

***Role of Age.*** A stepwise linear regression procedure was applied to investigate the effect of power and age on interoceptive accuracy. First, we regressed participants' interoceptive accuracy scores on power conditions (dummy coded) and age (mean-centered). The effect of age on interoceptive accuracy was not statistically significant ($t < 1$, $p = .46$). However, as expected, the effect of social power on interoceptive accuracy remained significant and in line with our proposition ($b_{\text{Powerful vs. Control}} = 12.42$, $SE_b = 4.69$, $t(127) = 2.65$, $p = .009$, 95% $CI_b$ [3.15, 21.70]; $b_{\text{Powerful vs. Powerless}} = 14.41$, $SE_b = 4.64$, $t(127) = 3.11$, $p = .002$, 95% $CI_b$ [5.24, 23.59]). Next, when we entered the respective interaction terms between power conditions (dummy coded) and age (mean-centered)

into the model, the interaction terms were not significantly predicting interoceptive accuracy (*ps* > .69). These analyses demonstrate that participants' age does not predict their interoceptive accuracy, nor does it moderate the relation between power and interoceptive accuracy.

***Role of Physical Exercise Frequency.*** Past findings suggest that participants who exercise more frequently show higher interoceptive accuracy (Cameron, 2001; Schandry & Bestler, 1995). To control for its effect in our findings, we measured participants' frequency of weekly exercises using a 5-point scale (1 = never, 2 = once a week, 3 = twice a week, 4 = three times a week, 5 = four times a week or more). First, to ensure that our power manipulation did not predict participants' reported exercise frequency, we ran a one-way ANOVA with power condition as an independent variable and participants' frequency of weekly exercises as a dependent variable. Results indicated that our manipulation did not significantly influence participants' reporting of their physical exercises ($F < 1$, $p = .63$).

Next, a stepwise linear regression procedure was applied to investigate the effect of power and exercise frequency on interoceptive accuracy. First, we regressed participants' interoceptive accuracy scores on power conditions (dummy coded), and exercise frequency (mean-centered). Results revealed that exercise frequency was positively and significantly associated with people's interoceptive accuracy ($b = 3.120$, $SE_b = 1.58$, $t(127) = 2.03$, $p = .045$, 95% $CI_b$ [0.07, 6.32]). However, as expected, the effect of social power on interoceptive accuracy remained significant and consistent with our proposition ($b_{\text{Powerful vs. Control}} = 11.79$, $SE_b = 4.63$, $t(127) = 2.55$, $p = .012$, 95% $CI_b$ [2.63, 20.95]; $b_{\text{Powerful vs. Powerless}} = 13.82$, $SE_b = 4.58$, $t(127) = 3.02$, $p = .003$, 95% $CI_b$ [4.75, 22.89]). Finally, we entered the respective interaction terms between power (dummy coded) and exercise frequency (mean-centered) into the model to investigate potential interaction effects.

However, results revealed that interaction terms were not significantly predicting interoceptive accuracy (*ps* $> .17$).

Together these results indicate that, irrespective of their experimental power conditions, participants who exercised more frequently were more accurate in perceiving their bodily signals. Importantly, however, the effect of power on interoceptive accuracy was robust and remained significant independent of participants' exercise frequency.

***Role of Task Involvement.*** Participants indicated their level of attentiveness during the experiment using a 7-point scale (from 1 = not at all attentive to 7 = very attentive). Results of a one-way ANOVA revealed that our power manipulation (high-power vs. low-power vs. control) did not have any influence on participants' self-reported attentiveness ($p = .87$). Moreover, a stepwise linear regression procedure was applied. First, we regressed participants' interoceptive accuracy scores on power conditions (dummy coded to compare high-power condition with control and low-power conditions), and self-reported task involvement (mean-centered). The effect of task self-reported involvement on interoceptive accuracy was not statistically significant ($p = .31$). However, we found a main effect of power on interoceptive accuracy consistent with our proposition ($b_{\text{Powerful vs. Control}} = 12.10$, $SE_b = 4.68$, $t(127) = 2.58$, $p = .01$, 95% $CI_b$ [2.83, 21.36]; $b_{\text{Powerful vs. Powerless}} = 14.54$, $SE_b = 4.62$, $t(127) = 3.15$, $p = .002$, 95% $CI_b$ [5.39, 23.69]). Next, we added the respective interaction terms between power and task involvement to our model. Results revealed that these interaction terms were not significantly predicting interoceptive accuracy (*ps* $> .88$).

***Records of Past or Current Heart Conditions.*** Participants were asked to indicate whether they had ever experienced any kind of heart condition, and if so, to specify its type. In total, nine people in our sample had experienced heart problems at some point in their lives (high blood pressure (n = 2), heart surgery (n = 1), anxiety (n = 1), slow heartbeats (n = 1), arrhythmia (n = 2), second tone (n = 1),

and heart murmur (n = 1)). Three of them were in the low-power, two in the control, and four in the high-power experimental conditions. It might be that participants with heart ailments would be more sensitive to their internal signals. Consequently, to provide a more conservative test of our hypothesis, we re-ran the key analyses reported in the main article by excluding these participants from the sample. These analyses revealed also significant results consistent with the main findings reported in our manuscript. Particularly, we found *a)* a significant main effect of power on interoceptive accuracy, *b)* a significant interaction effect between power and body consciousness on interoceptive accuracy, and finally, *c)* a significant effect of participants' chronic sense of power on interoceptive accuracy, similar to, and independent of, experimentally induced feelings of power. These analyses will be provided upon request.

**References**


Cameron, O. G. (2001). Interoception: The Inside Story—A Model for Psychosomatic Processes. *Psychosomatic Medicine, 63*(5), 697-710. Retrieved from http://www.psychosomaticmedicine.org/content/63/5/697.abstract

Galinsky, Gruenfeld, D. H., & Magee, J. C. (2003). From power to action. *Journal of Personality and Social Psychology, 85*(3), 453-466. doi:Doi 10.1037/0022-3514.85.3.453

Galinsky, A. D., Magee, J. C., Gruenfeld, D. H., & Whitson, J. A. (2008). Power Reduces the Press of the Situation: Implications for Creativity, Conformity, and Dissonance. *Journal of Personality and Social Psychology, 95*(6), 1450-1466. doi:Doi 10.1037/A0012633

Guinote. (2007). Power and goal pursuit. *Personality and Social Psychology Bulletin, 33*(8), 1076-1087. doi:Doi 10.1177/0146167207301011

Guinote, A., Judd, C. M., & Brauer, M. (2002). Effects of power on perceived and objective group variability: Evidence that more powerful groups are more variable. *Journal of Personality and Social Psychology, 82*(5), 708-721. doi:10.1037/0022-3514.82.5.708

Overbeck, J. R., & Droutman, V. (2013). One for All Social Power Increases Self-Anchoring of Traits, Attitudes, and Emotions. *Psychological Science, 24*(8), 1466-1476.

Schandry, R., & Bestler, M. (1995). The association between parameters of cardiovascular function and heartbeat perception. In D. Vaitl & R. Schandry (Eds.), *From the heart to the brain: The psychophysiology of circulation–brain interaction* (pp. 223–250). New York: Peter Lang.

Brener, J., Knapp, K., & Ring, C. (1995). The effects of manipulating beliefs about heart-rate on the accuracy of heartbeat counting in the Schandry task. *Psychophysiology* (Vol. 32, pp. S22-S22).
